# Influence of biased feedback on performance in a Vernier discrimination task

**DOI:** 10.3389/fpsyg.2022.987042

**Published:** 2023-01-13

**Authors:** Chenfan Yang, Ziran Xu, Yaoyao Zhong, Tianze Wang

**Affiliations:** ^1^Guangdong Business and Technology University, Zhaoqing, China; ^2^Faculty of Education, Guangxi Normal University, Guilin, China; ^3^Guangxi Colleges and Universities Key Laboratory of Cognitive Neuroscience and Applied Psychology, Guilin, China

**Keywords:** biased feedback, Vernier discrimination tasks, reversed feedback, sensory evidence, prior belief

## Abstract

The influence of feedback on performance is a topic of ongoing debate, with some previous studies finding it to be ineffective, while others have discovered that it can be helpful or harmful. One possible reason for these inconsistent results may be that feedback can create a conflict between a person's beliefs and the sensory information they receive. In the present study, we used a Vernier discrimination task to examine the influence of biased feedback on performance, as this type of feedback is most likely to create conflict. Biased feedback refers to feedback that does not align with the subjects' choices. The Vernier discrimination task is a type of psychophysical task that is often used to measure an individual's ability to perceive differences in the position or orientation of two visual stimuli. The task involves presenting two stimuli, one of which is slightly offset from the other, and asking the individual to determine the direction and magnitude of the offset. In Experiment 1, feedback was provided after each trial using large-offset verniers as guidance. The large-offset verniers always received correct feedback, but the small and medium-offset verniers might receive biased feedback. In Experiment 2, feedback was provided after each block of eight verniers. In Experiment 3, we removed the large offset vernier to investigate the influence of block feedback on the signal and noise. The results showed that the accuracy for the target vernier decreased due to biased feedback in both the trial feedback (Experiment 1) and the block feedback (Experiment 2). However, in Experiments 1 and 2, performance improved when feedback was absent. Moreover, if the difference between the two types of stimuli is great, the individual will engage in encoding learning rather than decision learning (Experiments 1 and 2). If the discrimination between the two types of stimuli is low, an individual's ability to discriminate noise is more vulnerable to the influence of biased feedback than the ability to discriminate the signal (Experiment 3). These results are discussed in relation to the mechanism of biased feedback, the process of encoding learning, the monitoring of internal feedback, and the generalization of false decisions.

## 1. Introduction

At its most basic level, feedback refers to information an individual receives about his/her past behavior. It provides some information about the correctness, accuracy, or adequacy of a response (Ilgen et al., 1979). Kluger and DeNisi (1996) defined “feedback intervention” as actions taken by external agents to provide information regarding some aspect(s) of one's task performance. Feedback can accurately reflect the results of a task (true feedback). However, it may also be incorrect (biased feedback) or irrelevant (fake feedback) to the results. Biased feedback refers to feedback that is reversed. For example, if the participant responds correctly, the feedback would be “incorrect” (Herzog and Fahle, 1999; Aberg and Herzog, 2012). Conversely, “fake feedback” refers to random feedback that is irrelevant to the response (Alloy and Abramson, 1979; Beedie et al., 2012).

The influence of feedback on performance is a matter of debate. Early studies on feedback intervention focused mainly on true feedback (Wright, 1906; Arps, 1920; Book and Norvell, 1922; Gilliland, 1925; Thorndike, 1927; Brown, 1932; Manzer, 1935; Elwell and Grindley, 1938), where subjects were given “correct” for correct responses and “incorrect” for incorrect responses. For example, Thorndike (1927) found that providing subjects with feedback on their results during a blindfold task could significantly improve accuracy. These studies covered a variety of fields, including muscle movement (Wright, 1906; Arps, 1920), perception of speed (e.g., Hill and Salzman, 2012; Molloy et al., 2018), executive function (e.g., Tarullo et al., 2018), and high-level mental functions such as reasoning (Book and Norvell, 1922). Different researchers have different theories, referring to attention (Arps, 1920), motivation (Book and Norvell, 1922; Manzer, 1935), organization of knowledge (Elwell and Grindley, 1938), psychological benefits (Wright, 1906), and so on. However, some studies found that feedback interventions can be ineffective (e.g., Locke, 1967; Locke and Bryan, 1969; Adams, 1978) and may even reduce performance (e.g., Katz et al., 2014). An overview by Kluger and DeNisi (1996) found that feedback intervention reduced performance in more than one-third of the studies they examined.

Similar to the influence of true feedback, the influence of fake feedback remains controversial. Fake feedback suggests that there is no relationship between feedback and real performance (random presentation). As for fake feedback, Hurlock's (1925) study is representative. He evaluated the effect of continuous blame and praise on children's arithmetic abilities over a period of time. Hurlock divided subjects into four groups and asked them to complete an arithmetic task after providing them with different types of feedback (regardless of their real performance). The praise group received praise and encouragement after every task. The criticism group was severely criticized after each task. The neglected group received neither blame nor encouragement and remained in the same classroom with the praise and criticism groups. The control group was separated from the other groups and received no feedback. Hurlock found that praise had the strongest effect on improving performance, which continued to increase over the course of the experiment. Initially, criticism had the same effect as praise, but its effect later decreased. The performance of the neglected group also improved at first but then declined significantly. The performance of the control group exhibited no change.

Fake feedback also triggers a variety of psychological processes. For example, in terms of visual perception, Phillips et al. (1999) presented subjects with fake heart rate information and then asked them to detect whether their heartbeats and a flash of light appeared simultaneously. The results showed that fake slow feedback decreased accuracy. In terms of duration perception, Chwilla and Brunia (1991) found that, after receiving fake feedback, subjects overestimated the duration of the stimuli. Schwark et al. (2012) and Rigoni et al. (2016) observed the effects of fake feedback on visual stimuli and response inhibition, respectively. Gray et al. (2007) found that, at an emotional level, fake feedback could improve emotional perception. Beedie et al. (2012) found that, when cyclists received feedback that their riding time was shorter than that of others, their happiness and calmness improved, while their anxiety, depression, inertia, and other negative emotions were worse than those of the control group. In addition, fake feedback also had an impact on their physiological health. In the same experiment, fake feedback also reduced oxygen consumption and improved blood glucose levels. The findings of McCall and Meston (2007) also confirmed the effect of fake feedback on their physiological health. They found that fake positive feedback could improve one's sexual arousal. Thus, fake feedback may also improve or reduce individual performance, similar to true feedback.

The different effects of feedback (both true and fake) may be related to perceptual factors at different stages of the decision-making process, from the initial perception of stimuli to the final decision. The conflict between different stages may determine how feedback works. Herzog and Fahle (1999) divided the decision-making process into three stages: early, middle, and late. The early stage involves the initial perception of stimuli, such as the gap between two line segments. However, individuals may perceive a complete line segment in the early stages if the gap is significantly small. The intermediate stages are related to factors that are not linked with the properties of the stimulus, such as task requirements, warnings, feedback, and other information. They might lead to an unstable representation of the environment, appearing unpredictable and potentially threatening (Varrier et al., 2019). At the late stage (decision stage), individuals need to comprehensively evaluate information from the early and intermediate stages and finally make decisions. At this stage, the weight of the early and middle stages is the decisive factor. The factors in the middle stage are measurable and controllable. However, the sensory information individuals perceive in the early stage is not always consistent with the final behavior results; thus, it is hidden and difficult to measure. It is unclear how sensory information interacts with prior beliefs that are triggered by feedback. We need to find a method to investigate the perception of sensory information. Sensory information refers to the subject's perception of the correctness of the results when there is no feedback (internal feedback in the present study).

Which kind of feedback is most effective for exploring the role of internal feedback? Biased or reverse feedback (Herzog and Fahle, 1999) is particularly useful in this regard (see Aberg and Herzog, 2012), as it provides “wrong” feedback when individuals make correct judgments or “right” feedback when they make wrong judgments. This kind of feedback will create conflicts between sensory information and prior beliefs. At present, there are few studies on biased feedback, and some researchers call false feedback “fake feedback” in essence (e.g., Story and Craske, 2008). One way to control feedback is to change its prevalence (Wolfe and Van Wert, 2010). Prevalence refers to the probability of biased feedback in a group involving true feedback. Increasing the prevalence of biased feedback can lead to greater uncertainty, influencing the subject's perception and judgment criteria.

To control sensory information, studies can manipulate the offset of verniers in Vernier discrimination tasks. In a vernier discrimination task, two straight lines (called verniers) appear above and below each other, and the two verniers may be aligned or offset. The subject needs to determine whether they are offset by pressing different keys. Herzog and Fahle (1999) found that biased feedback would decrease individuals' performance in Vernier discrimination tasks, but once the feedback became correct, the performance would improve. This study provided a basic hypothesis: biased feedback will decrease performance.

However, it is important to note that the measurement of sensory information may not always accurately reflect an individual's actual perception. In the study by Herzog and Fahle (1999), verniers with different offset levels were used, and the values of the medium- and small-offset levels were two-third and one-third of the large offset level, respectively. However, it is well-known that sensory threshold is not linearly related to the physical properties of stimuli. Thus, verniers at the three levels might not have been distinguishable to the subjects. The subjects' perception of the three verniers may not have necessarily followed the 3:2:1 ratio. Therefore, the perception of the verniers may be different from the 3:2:1 ratio. For example, with the logic of Herzog and Fahle (1999), the number of offsets perceived by subjects in the medium cursor should be two-third of the number in the large cursor. However, in reality, the number the subjects perceive is likely less than this value because of the non-linearity of the sensory threshold. Thus, the prior sensory information may not be consistent with the actual perception.

Another problem was that the effect of the offset was likely to be related to the difficulty of the task. The greater the offset of two verniers, the lower the difficulty. In Herzog and Fahle's (1999) study, medium-offset verniers were easier to identify than small-offset verniers. Although they found that the accuracy of medium-offset verniers was higher, this may be due to the task's difficulty. The offsets of the three levels of verniers were noticeably similar, and these levels were presented in a mixed manner in each block. It was difficult to discern whether difficulty played a role in their study.

**Figure 1:**
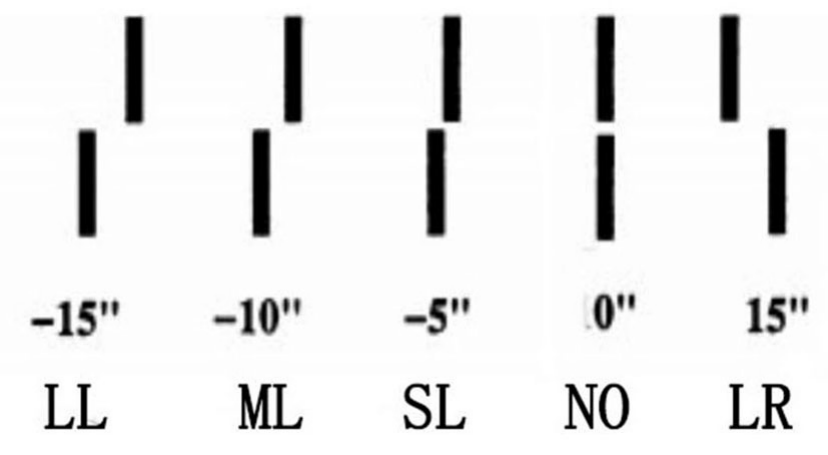
An example of offsets. From left to right are the conditions of large left offset (LL), middle left offset (ML), small left offset (SL), no offset (aligned verniers, NO), and large right offset (LR), respectively.

We thus designed three experiments to explore the impact of the conflict between sensory information (internal feedback in the present study) and feedback (called external feedback in the present study) on the performance of Vernier discrimination tasks. In Experiment 1, we referred to the setup of Herzog and Fahle (1999) and asked subjects to judge whether two verniers were offset. However, to avoid the insufficient discrimination caused by mixed offset levels, we set the offset level as a between-group factor (offset size: medium vs. small), which was originally a within-group factor, as in the study by Herzog and Fahle (1999). Half of the trials in each block used large-offset verniers as guidance. In the pre-experiment, the large offset level always achieved 100% accuracy. The other half of the trials involved medium- or small-offset verniers in different groups. The size of the medium-offset vernier was no longer two-thirds of the large offset level but still corresponded to an accuracy of 85–95% in the pre-experiment. The size of the small offset vernier corresponded to an accuracy of 55–65%. Figure 1 shows an example of the offset levels used in the study. The large-offset verniers would prevent the subjects from perceiving too much biased feedback and giving reverse responses. This ensured that the verniers were presented under the “wings” of correctly labeled and easily discernible verniers (Herzog and Fahle, 1999).

Another important variable was the probability of biased feedback (high vs. low). In the high group, the probability of biased feedback on target verniers was 80%, while it was 20% in the low group. Biased feedback appeared only after medium- and small-offset verniers. In Experiment 1, we predicted that both offset size groups would be influenced by biased feedback, while the large-offset verniers would not be influenced because they received no biased feedback. The performance with large-offset verniers may reflect the presence of decision learning. Herzog and Fahle (1999) divided feedback-based learning into encoding learning (mislearning) and decision learning (misdecision). In encoding learning, feedback might act as a classifier, providing the associated class label. False data labeling might lead to a misclassification based on a change in likelihood functions. Feedback might also influence the decision-making process. For example, if feedback reinforces one class of decision over the other, a shift in the decision criteria might take such biased feedback into account. A shift in the decision criterion yields more responses toward the favored side. All these changes related to decision processes occur after the encoding stages. Herzog and Fahle (1999) found that, even if biased feedback followed only the small-offset verniers, it influenced large, medium, and small verniers at the same time. Based on these results, they argued that there was a misdecision rather than mislearning. Otherwise, the biased feedback would influence only the small-offset verniers. We made the opposite prediction. If the three types of verniers were not mixed (between groups) and there was sufficient discrimination among them, mislearning would be likely to occur. Subjects would mistakenly classify the small left verniers as right, but the large-offset verniers would not be influenced in the high group.

In the trial feedback of Experiment 1, we found that difficulty and biased feedback decreased the subjects' performance. The average accuracy of different offset sizes indicated the degree of difficulty. A medium offset is easier to distinguish compared with a small offset. The accuracy of different types of feedback indicated the effect of feedback. In the trial feedback, the subjects could clearly see whether they were right or not, having received clear internal feedback. However, group (block) feedback could be different. In a group feedback study by Aberg and Herzog (2012), they presented block feedback after every seven trials and did not find any feedback effect. This may be because subjects did not assess their correctness and therefore did not generate clear internal feedback; thus, there was no conflict between internal and external feedback, resulting in no effect of the biased feedback. We asked participants to evaluate their performance after every eight trials (a block) and then gave consistent or biased feedback. That is, subjects must first estimate how many of the eight trials they got right after each block, and then, the program will give them consistent or inconsistent feedback based on their estimates. Subjects' estimates would generate internal feedback. When their internal feedback conflicts with external feedback, biased feedback may decrease performance. Experiments 2 and 3 in the present study explored the influence of block feedback. Subjects were required to evaluate their accuracy after each block, and biased feedback was presented according to their evaluated accuracy. Experiments 2 and 3 directly set up feedback that was opposite to the self-assessment to explore the role of biased feedback.

Experiment 2 found that block feedback could influence performance. In Experiment 3, there were no large-offset verniers because there were few indicators with such a large distinguishability as guidance in real life. In Experiment 3, subjects were no longer asked to judge the direction of the offset but to judge whether the two verniers were offset. Half of the verniers were offset, while the other half were aligned in Experiment 3. This would help us explore the influence of signal (offset verniers) and noise (aligned verniers). It is worth noting that, in Experiments 2 and 3, we set up three completely correct feedback blocks before the occurrence of biased feedback, which always provided feedback based on real performance.

This study is novel in that it aims to clarify how sensory information interacts with prior beliefs triggered by feedback. The prior beliefs triggered by feedback mean that individuals would expect later trials when they received feedback in previous trials. This belief may change along with the presentation of feedback. In Experiment 1, we tested mislearning and misdecisions when the verniers were more distinguishable. We also tested the influence of difficulty. In Experiments 2 and 3, we tested block feedback based on the subjects' evaluations. Currently, there are only a few studies on biased feedback, mostly based on real results rather than the subjects' evaluations.

In summary, we put forward the following hypotheses:

1. Offset size (difficulty) would influence accuracy, but biased feedback would also decrease performance when excluding the influence of difficulty.
2. Biased feedback would decrease performance regardless of whether there was trial feedback or block feedback.
3. When biased feedback disappeared, performance would rebound.
4. The performance of large-offset verniers would not decrease when we provided biased feedback for small and medium-offset verniers, that is, misreading rather than misdecision would occur.

## 2. Experiment 1: Trial feedback

### 2.1. Materials and methods

#### 2.1.1. Participants

Herzog and Fahle (1999) recruited five subjects for each condition. In the study by Aberg and Herzog (2012), six subjects were recruited for each condition. Both studies found bias feedback effects, but neither reported effect size. Thus, we could not calculate the sample size *via* a priori power analysis with any existing effect size. To ensure that the effects of biased feedback could be observed, 12 participants were recruited at each level, which was exactly two times the sample size of Aberg and Herzog (2012). A total of 48 students (mean age = 21.04 years, 20 men, 28 women) were randomly divided into four groups. All participants in the present study (all three experiments) were recruited from the Guangxi Normal University, in the same major and grade, with normal or corrected-to-normal vision. During recruitment, they were informed that they would receive fixed rewards. None of our participants participated in more than one experiment. All experiments were approved by the ethics committee of the Ministry of Education at Guangxi Normal University. The participants provided their written informed consent to participate in the study. After the experiments, we informed each subject of the present study's purpose: to eliminate the influence of feedback intervention.

#### 2.1.2. Stimuli

The experiment was conducted using E-Prime 2.0 software on a computer with a 21-inch monitor with a resolution of 1,920 × 1,080 and a refresh rate of 65 Hz at a distance of 1 m. Verniers consisted of two vertical lines with a length of 7.18′ (arcmin), separated by a gap of 0.72′. The lower line was offset to the left or right in relation to the upper line. The luminance was approximately 200 cd/m. Based on our pre-experiment results, each trial's presentation time was set to 50 ms, during which the offset of the verniers was highly distinguishable.

We determined the appropriate offset level by measuring the offset threshold for each participant in the pre-experiment. Herzog and Fahle (1999) decided that the values of the medium- and small-offset levels were two-thirds and one-third compared with the large-offset level, respectively. However, in reality, the number the subjects perceive is likely to be less than this value because of the non-linearity of the sensory threshold. Thus, in the present study, we set different offset levels to measure the subjects' accuracy. Subjects needed to distinguish whether a vernier was offset to the right or the left. The large offset condition corresponded to the 100% threshold level. The subjects could always respond correctly to the verniers of this condition in the 20 trials of a block presented in the pre-experiment. For the measurement of large offset verniers, an increasing sequence was used. Every 20 trials represented a block. The offset increased with the block until subjects obtained 100% accuracy. The medium-offset condition corresponded to the threshold level of 85–95%. The subjects had an 85–95% probability of a correct response. The small offset condition corresponded to the 55–65% threshold level. The decreasing sequence was used at these two levels, starting from the offset pertaining to the large-offset verniers until they first reached the abovementioned accuracy.

#### 2.1.3. Task and procedure

For each stimulus, the offset and condition of feedback could be manipulated independently. There were two levels of offset size (medium vs. small) and probability of biased feedback at two levels (high vs. low). In each trial, two verniers were presented in the center of the screen, and participants indicated whether the lower line was offset to the left or the right relative to the upper line by pushing one of the two buttons.

There were 12 blocks in Experiment 1, and each block consisted of 20 trials. In each block, 10 trials presented large offset verniers, while the other 10 presented medium-offset verniers (in the medium group) or small-offset verniers (in the small group). For large-offset verniers, half of the lower lines were offset to the left, while the other half were offset to the right. Large-offset trials always received true feedback. For the medium- (in the medium group) and small-offset verniers (in the small group), all the lower lines were offset to the left. Only the medium (small) offset verniers would receive different feedback. In the low group, the medium (small) trials had a probability of 20% to meet reverse feedback and a probability of 80% to meet true feedback. In the high group, the medium (small) trials had a probability of 80% to meet reverse feedback and a probability of 20% to meet true feedback. True feedback involved giving “incorrect” feedback when the subjects made incorrect judgments. Reverse feedback provided “incorrect” feedback when the subjects made correct judgments. No “correct” feedback would be given, similar to the work by Herzog and Fahle (1999).

The first seven blocks were feedback blocks that appeared immediately after the subjects responded. The last five blocks were rebound blocks, in which there was no feedback. There was an introduction between the 7th and 8th blocks to inform the subjects that there would be no more feedback.

### 2.2. Results

#### 2.2.1. The accuracy of key blocks

First, we calculated the difference (change amount) in the accuracy of the first and seventh blocks. The target offset size (medium vs. small) and the probability of biased feedback (high vs. low) were within-group variables. However, we also tested the large-offset verniers to calculate whether there was a misdecision or mislearning. If the biased feedback impacted both the large-offset verniers and the target verniers (the middle offset in the middle group and the small offset in the small group), it would imply a misdecision. If biased feedback only impacted the target vernier, it would imply mislearning. We set the vernier type (big left vs. target vs. big right) as a within-group variable. A mixed ANOVA was performed with the variables of offset size, feedback type, and vernier type. For the vernier type, the target in the small group involved a small left vernier, while it was the medium left vernier in the medium group.

**Figure 2:**
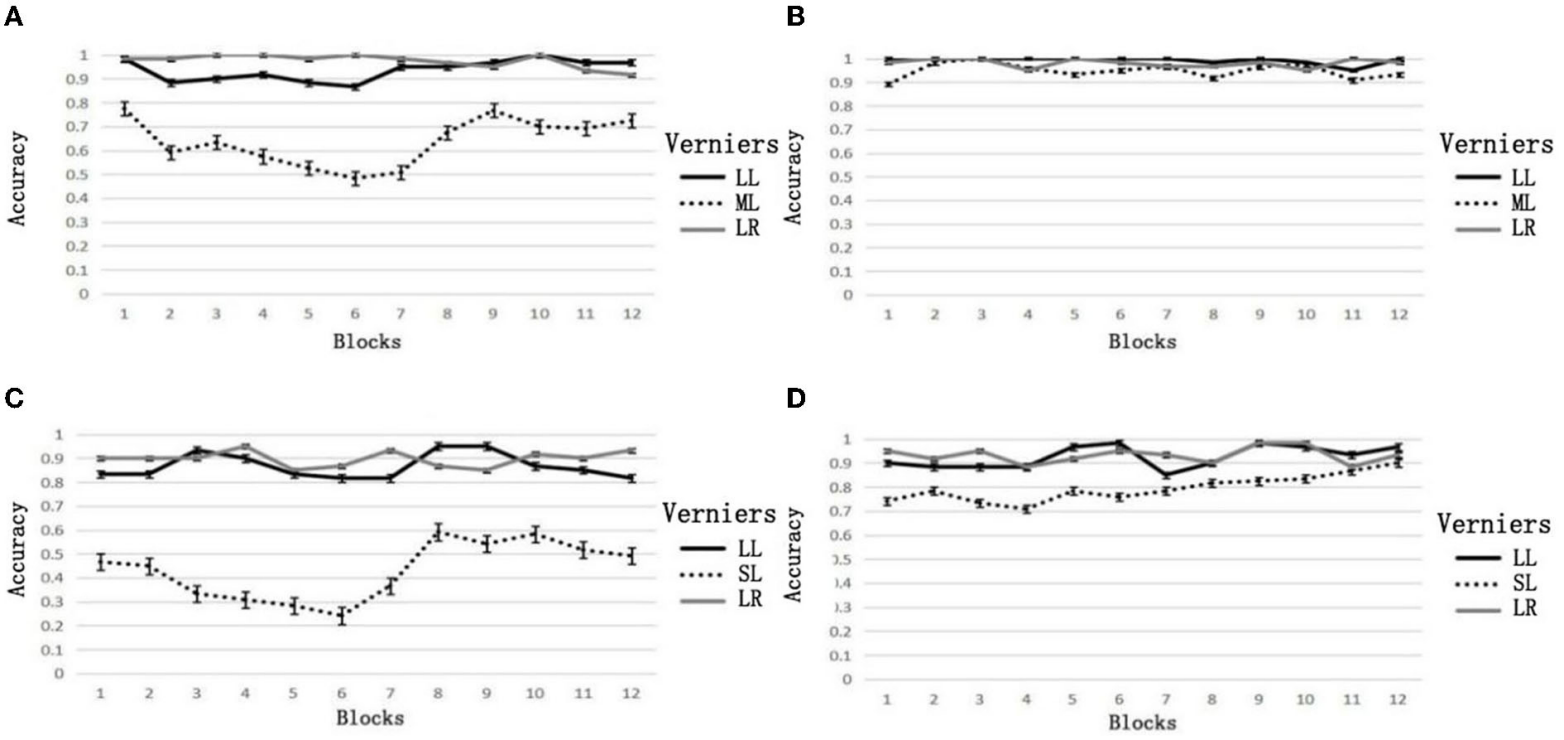
Accuracy of trial feedback. **(A)** Medium, biased feedback, **(B)** medium, consistent feedback, **(C)** small, biased feedback, **(D)** small, consistent feedback.

The results showed that only feedback type was marginally significant for the main effects, *F*_(1,44)_ = 4.057, *p* = 0.05, $\eta_{\text{p}}^{2}$ = 0.084. The change in the low group (−0.006 ± 0.145) was less than that in the high group (0.064 ± 0.222). This indicated that the accuracy of the high group decreased more from the first block to the seventh block than that of the low group. For the interaction effects, only the feedback and vernier types had a significant difference; *F*_(2,88)_ = 10.391, *p* < 0.001, $\eta_{\text{p}}^{2}$ = 0.191. The simple effect showed that the change of the target (0.183 ± 0.263) in the high group was significantly greater than that of the large left vernier (0.025 ± 0.223) and large right vernier (−0.017 ± 0.101), *p*_*s*_ ≤ 0.006. There was no difference between the large left vernier and large right vernier; *p* = 0.121. There was no difference in the low group; *p*_*s*_ ≥ 0.121. There was no difference in any other main effects or interactions, *p*_*s*_ ≥ 0.154, $\eta_{\text{p}}^{2}$ ≤ 0.046. These results indicated that biased feedback decreased targets' accuracy but did not affect large verniers. Besides, the low group had no effect on reducing or improving accuracy. In addition, the influence of biased feedback was not affected by the offset size (see Figure 2).

Since biased feedback influenced only the targets, we tested the differences in the targets in the first, seventh, and twelfth blocks in the low group to explore how the accuracy of the target was reduced and improved under different conditions. These three blocks were taken because the first block served as a baseline, the seventh block had the last block influenced by feedback, and the twelfth block marked the end of the accuracy rebound. However, in the high group, the accuracy of the targets reached its lowest point in the sixth block; thus, we tested the accuracy of the first, sixth, and twelfth blocks.

The results showed that, for the middle offset vernier, the accuracy of the high group decreased from 0.775 ± 0.176 in the first block to 0.483 ± 0.301 in the sixth block and rebounded to 0.725 ± 0.328 in the twelfth block. For the small-offset verniers, the accuracy of the high group decreased from 0.467 ± 0.284 in the first block to 0.242 ± 0.178 in the sixth block and rebounded to 0.492 ± 0.257 in the twelfth block. In the low group, the accuracy of the medium-offset vernier in the first, seventh, and twelfth blocks was 0.892 ± 0.173, 0.967 ± 0.049, and 0.933 ± 0.123, respectively. The accuracy of the small-offset verniers in the three blocks was 0.742 ± 0.261, 0.783 ± 0.252, and 0.900 ± 0.141, respectively. We compared the accuracy in the sixth and twelfth blocks to test how individuals rebound in terms of learning a good or bad prior and then correcting themselves with sensory information. The *t*-test results are shown in Table 1.

**Table 1:** *T*-tests of trial feedback.

**Feedback type**	**Offset size**	**Blocks**	**Mean difference**	***t***	***p***	***d***
Consistent feedback	Small	1 vs. 7	−0.417	−0.594	0.564	0.239
		7 vs. 12	−0.117	−1.606	0.137	0.630
		1 vs. 12	−0.158	−2.048	0.065	0.793
	Medium	1 vs. 7	−0.075	−1.517	0.157	0.598
		7 vs. 12	0.033	1.076	0.305	0.429
		1 vs. 12	−0.042	−0.671	0.516	0.270
Biased feedback	Small	1 vs. 6	0.225	2.265	0.045	0.868
		6 vs. 12	−0.250	−4.855	0.001	1.548
		1 vs. 12	−0.025	−0.208	0.839	0.084
	Medium	1 vs. 6	0.267	4.222	<0.001	1.184
		6 vs. 12	−0.217	−2.091	0.05	0.847
		1 vs. 12	0.05	0.736	0.477	0.296

#### 2.2.2. The average accuracy

We tested the influence of difficulty by comparing the average accuracy of all blocks of the small and medium-offset verniers under different types of feedback. The results showed that the main effects of feedback types were significant, *F*_(1,44)_ = 45.015, *p* < 0.001, $\eta_{\text{p}}^{2}$ = 0.506. The mean accuracy of the low group (0.827 ± 0.150) was higher than that of the high group (0.534 ± 0.230). There was a significant difference in the main effect of the vernier type; *F*_(1,44)_ = 12.860, *p* = 0.001, $\eta_{\text{p}}^{2}$ = 0.226. The mean accuracy of the medium-offset vernier (0.793 ± 0.228) was higher than that of the small-offset vernier (0.613 ± 0.256). There was no significant difference in the interaction; *F*_(1,44)_ = 0.269, *p* = 0.607, $\eta_{\text{p}}^{2}$ = 0.006. These results indicated that difficulty and feedback type influenced accuracy.

We also tested the average accuracy in different phases. The results were similar to the overall accuracy for the feedback phase (blocks 1–7). The main effects of feedback types were significant, *F*_(1,44)_ = 39.025, *p* < 0.001, $\eta_{\text{p}}^{2}$ = 0.470. The mean accuracy of the low group (0.905 ± 0.133) was higher than that of the high group (0.691 ± 0.143). There was a significant difference in the main effect of the vernier type; *F*_(1,44)_ = 17.951, *p* < 0.001, $\eta_{\text{p}}^{2}$ = 0.290. The mean accuracy of the medium–offset vernier (0.871 ± 0.142) was higher than that of the small offset vernier (0.725 ± 0.176). There was no significant difference in the interaction; *F*_(1,44)_ = 0.072, *p* = 0.789, $\eta_{\text{p}}^{2}$ = 0.002.

The results were similar to the overall accuracy for the rebound phase (blocks 8–12). The main effects of feedback types were significant, *F*_(1,44)_ = 20.819, *p* < 0.001, $\eta_{\text{p}}^{2}$ = 0.321. The mean accuracy of the low group (0.928 ± 0.098) was higher than that of the high group (0.778 ± 0.142). There was a significant difference in the main effect of the vernier type; *F*_(1,44)_ = 7.819, *p* = 0.008, $\eta_{\text{p}}^{2}$ = 0.149. The mean accuracy of the medium-offset vernier (0.898 ± 0.112) was higher than that of the small-offset vernier (0.807 ± 0.157). There was no significant difference in the interaction; *F*_(1,44)_ = 0.692, *p* = 0.410, $\eta_{\text{p}}^{2}$ = 0.015. These results indicated that, even though the performance recovered when there was no more feedback, the influence of biased feedback still lasted a while.

### 2.3. Discussion

Similar to the study by Herzog and Fahle (1999), we found that biased feedback decreased the accuracy of the discrimination task. However, there was no relationship between feedback type and offset size. These results indicate that the lower accuracy in the small group was due to the greater difficulty of identifying small-offset verniers rather than the impact of biased feedback. If the latter were the case, we would expect to see an interaction between feedback type and offset size. These results may imply that the effect of biased feedback follows an all-or-nothing rule rather than decreasing with the increase in the discrimination of the offset.

In addition, we observed no influence of biased feedback on the large offset verniers. In the study by Herzog and Fahle (1999), the three verniers were mixed and barely distinguishable. This might be the reason why the accuracy of all three verniers was influenced by biased feedback in their experiment. In the present study, we found that if the large offset vernier and the target were significantly distinguishable, the subjects could resist the influence of biased feedback. In other words, the effect of biased feedback was not generalized. In addition, based on the logic of Herzog and Fahle (1999), since the large offset vernier was not affected, it could be considered that encoding learning rather than decision learning occurred in the present study. The subjects mistakenly classified the targets rather than making incorrect decisions for all left verniers.

Moreover, we tested the effect of difficulty (offset size). We found that the difficulty influenced the performance of the vernier discrimination task. However, biased feedback also decreased performance. In addition, Herzog and Fahle (1999) provided true feedback in the last five blocks and observed the rebound effect. We found that the influence of encoding learning could be eliminated without any feedback. In our experiment, once the biased feedback disappeared, even if there was no true feedback, just like in the study of Herzog and Fahle (1999), the accuracy was restored. Thus, true feedback is not a requirement for the recovery of performance.

In Experiment 1, the offset size did not always correspond to the internal feedback of the subjects. For example, in the middle offset group, the subjects should have had an accuracy of 85–95%, but this did not mean that they realized they had such accuracy in a block. It should be noted that the subjects' judgment of accuracy was based on several trials. For example, they might think 18 of the 20 trials were correct instead of the probability of one single trial. The probability of one single trial would have only two values: 0 or 1. Therefore, we used block feedback to directly explore the relationship between internal feedback and external feedback in Experiment 2. Subjects were asked to evaluate the correct number of eight trials in each block, and feedback was provided accordingly. This method corresponds to the work by Aberg and Herzog (2012). Unlike their experiments, we removed the medium-offset level, and feedback was provided based on the subjects' reports rather than the real results. This method also fixed the task's difficulty aspect.

## 3. Experiment 2: Block feedback

### 3.1. Materials and methods

#### 3.1.1. Participants

A total of 24 students (mean age = 21.12 years, 11 men, 13 women) were randomly divided into two groups.

#### 3.1.2. Stimuli

Same as in Experiment 1.

#### 3.1.3. Task and procedure

Similar to the study by Aberg and Herzog (2012), there were 15 blocks, and each block consisted of eight trials. The first three blocks were the practice blocks, the fourth to tenth blocks were the feedback blocks, and the tenth to fifteenth blocks were the rebound blocks. There was an introduction between the 10th and 11th blocks to inform the subjects that there would be no more feedback. Each block presented four large-offset verniers, including two offsets to the left and two to the right. The other four verniers were small-offset verniers, and all were offset to the left. After each block, an evaluation window appeared, which asked the subjects to evaluate how many of the eight trials were answered correctly.

For practice blocks, feedback was the same as the number of subjects' correct responses. For rebound blocks, there was no feedback. The feedback block provided feedback according to the subjects' evaluations. That is, subjects must first estimate how many of the eight trials they got right after each block, and then the program will give them consistent or inconsistent feedback based on their estimates. For example, in the consistent group, if participants answered they had seven correct responses in a block, the program would give “good” feedback such as “You have made eight correct judgments.” In the biased group, they would get “bad” feedback such as “You have made five correct judgments.” Since each block consisted of two parts, according to the results of the pre-experiment and Experiment 1, the accuracy of the large-offset verniers was always close to 100%. Assuming that the responses of the large-offset verniers are always correct, the total accuracy should range from four to eight. If the accuracy was <5 in any block, the experiment would be terminated because this implied that the subjects had no confidence in their responses regarding the small-offset verniers. However, no subject's experiment was terminated.

We partly reversed the accuracy of the small-offset verniers in the feedback blocks. For the high group, when the number of correct trials evaluated by the subjects was five or six, they would receive feedback that they had correctly answered seven or eight trials randomly. When the number of correct trials evaluated by the subjects was seven or eight, the feedback would be five or six trials at random. That is, when self-reported accuracy was low, subjects would receive feedback with high accuracy, and vice versa. For the low group, when the number of correct trials evaluated by the subjects was five or six, the feedback would be five or six trials at random. When the number of correct trials evaluated by the subjects was seven or eight, the feedback would be seven or eight trials at random. This design was implemented to maintain balance with the subject's sense of self and to prevent them from realizing that there was something wrong with the feedback. We did not consider the correct number below four because each block had four large-offset verniers, and their accuracy should be 100%. If the correct number of feedback was <4, the subject might realize that there was a problem with the feedback. At first, we were worried that the range of five to eight might be insufficient to observe the effect of biased feedback; however, the results showed such an effect.

### 3.2. Results

Since the large-offset verniers were only used as a guide, many subjects identified the large-offset verniers correctly in some blocks and had a derivation standard error of 0. We only analyzed the small-offset verniers. There was a practice effect on the task. The accuracy of the first and second block in the high group increased from 0.771 ± 0.328 to 0.979 ± 0.072, with a significant difference; *t*_(11)_ = 2.419, *p* = 0.034, *d* = 0.971. The low group's accuracy increased from 0.813 ± 0.155 to 0.938 ± 0.113, with a significant difference; *t*_(11)_ = 3.317, *p* = 0.007, *d* = 1.281.

**Figure 3:**
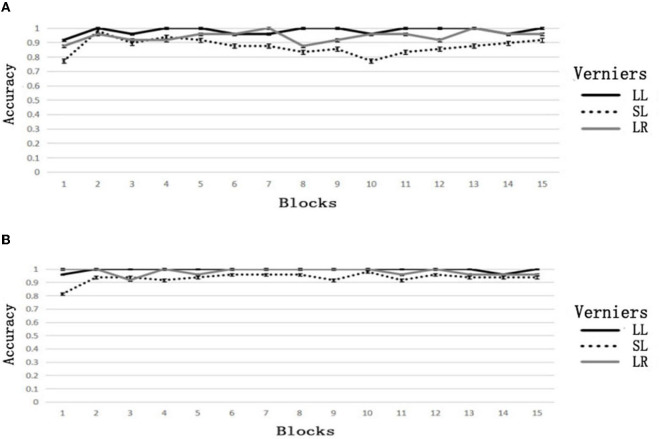
Accuracy of block feedback. **(A)** Biased feedback, **(B)** consistent feedback.

Paired sample *t*-tests were performed to analyze the accuracy between the low and high groups in the second, tenth, and fifteenth blocks. These three blocks were chosen because the subjects had practiced with the first block; thus, the second block was the beginning stage of familiarity. The tenth block was the last block influenced by feedback, and the fifteenth block was the end of the rebound. The accuracy of the high group decreased from 0.979 ± 0.072 in the second block to 0.771 ± 0.271 in the tenth block and rebounded to 0.917 ± 0.163 in the fifteenth block. The accuracy of the low group in these three blocks was 0.938 ± 0.113, 0.979 ± 0.072, and 0.938 ± 0.113, respectively. The change in accuracy is shown in Figure 3. The results of the paired sample *t*-tests are shown in Table 2.

**Table 2:** *T*-tests of block feedback.

**Feedback type**	**Blocks**	**Mean difference**	***t***	***p***	***d***
Consistent feedback	2 vs. 10	−0.042	−1.000	0.339	0.399
	10 vs. 15	0.042	1.000	0.339	0.399
	2 vs. 15	0.000	0.000	1.000	0.000
Biased feedback	2 vs. 10	0.208	2.803	0.017	1.054
	10 vs. 15	−0.146	−2.244	0.046	0.863
	2 vs. 15	0.062	1.915	0.082	0.744

### 3.3. Discussion

In Experiment 2, each block consisted of eight trials, while in the study by Aberg and Herzog (2012), each block had seven trials. The eight trials in our experiment were designed to balance the number of large and small-offset verniers. We did not include more trials because doing so might have lengthened the cycle of stimulus-feedback stimuli, resulting in ambiguous internal feedback. This is different from Aberg and Herzog's (2012) study, whose feedback was based on real performance and showed no effect of block feedback. We found that block feedback decreased performance. This indicated that the influence of external feedback depended more on whether it conflicted with internal feedback than on whether it affected real performance. In everyday life, we often need to estimate our own performance, such as when estimating the weight of vegetables at the store. The estimation might differ from the actual weight displayed on the scale, which could sometimes be due to overestimation or underestimation or, in some cases, dishonest tempering with the scale (which involves dishonest operation). These discrepancies may affect our future estimates. Experiments 1 and 2 proved that guidance, such as large-offset verniers, cannot prevent the mislearning of small-offset verniers. In real-life situations, there is sometimes no guidance when individuals make perceptual judgments. Therefore, we removed the large-offset verniers in Experiment 3 and asked the subjects to judge whether two verniers were offset or not. This could help us explore how the subject made trade-offs between the signal (offset verniers) and noise (aligned verniers) in the absence of guidance.

## 4. Experiment 3: Block feedback without guidance

### 4.1. Materials and methods

#### 4.1.1. Participants

A total of 24 students (mean age = 20.83 years, 12 men, 12 women) were randomly classified into two groups.

#### 4.1.2. Stimuli

Large-offset verniers were deleted. There were two types of verniers in Experiment 3. For offset verniers, the lower lines were always offset to the left with the small size. For aligned verniers, there was no offset. Each block consisted of four offset verniers and four aligned verniers.

#### 4.1.3. Task and procedure

Same as in Experiment 2, except for two settings: (A) Subjects were asked to judge whether there was an offset. (B) The feedback in the feedback blocks was different from Experiment 2. For the high group, when the number of correct trials evaluated by the subjects was zero to four, the feedback would be five to eight trials at random. When the number of correct trials evaluated by the subjects was from five to eight, the feedback would be zero to four trials at random. For the low group, when the number of correct trials evaluated by the subjects was zero to four, the feedback would be zero to four trials at random. When the number of correct trials evaluated by the subjects was five to eight, the feedback would be five to eight trials at random.

### 4.2. Results

The accuracy was close to the opportunity level of 0.5 when we deleted the large offset verniers. The mean accuracy in the low group was 0.522 ± 0.036, while it was 0.582 ± 0.141 in the high group. There was no practice effect. The accuracy of the first and second blocks in the low group was 0.542 ± 0.179 and 0.635 ± 0.155, respectively. The difference was not significant; *t*_(11)_ = 1.241, *p* = 0.241, *d* = 0.515. In the high group, there were 0.563 ± 0.229 and 0.656 ± 0.161, respectively. There was no significant difference; *t*_(11)_ = 1.750, *p* = 0.108, *d* = 0.718. Thus, the practice effect in Experiment 2 was meant to learn the judgments of large offset verniers.

Unexpectedly, the accuracy decreased even in the low group, from 0.635 ± 0.155 in the second block to 0.427 ± 0.164 in the tenth block. The accuracy of the fifteenth block (0.448 ± 0.125) did not rebound. The change in accuracy in the high group was similar to that in the low group, dropping from 0.656 ± 0.161 in the second block to 0.490 ± 0.247 in the tenth block. The accuracy of the fifteenth block (0.583 ± 0.215) did not rebound. The results of the *t*-tests are shown in Table 3.

**Table 3:** *T*-tests of block feedback without guidance.

**Feedback type**	**Blocks**	**Mean difference**	***t***	***p***	***d***
Consistent feedback	2 vs. 10	0.208	3.35	0.006	1.241
	10 vs. 15	−0.021	2.462	0.732	0.142
	2 vs. 15	0.188	−0.352	0.032	0.935
Biased feedback	2 vs. 10	0.167	2.966	0.013	1.103
	10 vs. 15	−0.094	−1.682	0.121	0.659
	2 vs. 15	0.073	1.168	0.267	0.465

**Figure 4:**
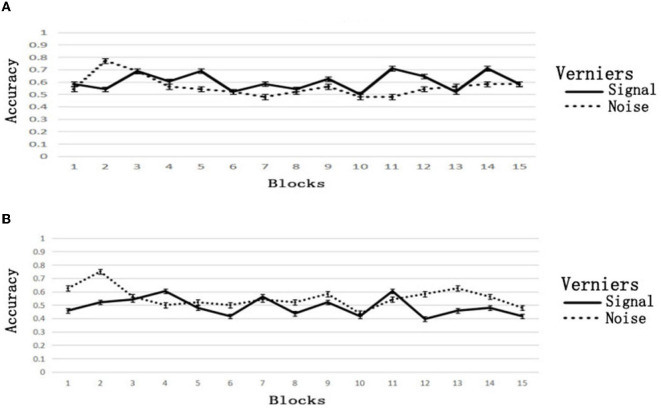
Accuracy of block feedback without guidance. **(A)** Biased feedback, **(B)** consistent feedback.

The decrease in accuracy came from the incorrect judgment of noise (aligned verniers). In the low group, the accuracy of the aligned verniers decreased from 0.750 ± 0.302 in the second block to 0.438 ± 0.304 in the tenth block. The accuracy of the fifteenth block (0.479 ± 0.391) did not rebound. The signal accuracy (offset verniers) in these three blocks was 0.521 ± 0.345, 0.417 ± 0.444, and 0.417 ± 0.359, respectively. Similar to the low group, the accuracy of the aligned verniers in the high group decreased from 0.771 ± 0.345 in the second block to 0.479 ± 0.391 in the tenth block, and the accuracy of the fifteenth block (0.583 ± 0.343) did not rebound. The accuracy of the signal in these three blocks was 0.542 ± 0.257, 0.500 ± 0.354, and 0.583 ± 0.404, respectively. The changes in accuracy are shown in Figure 4. The results of the *t*-tests of signals and noises are shown in Table 4.

**Table 4:** *T*-tests of signals and noises.

**Feedback type**	**Signal vs. noise**	**Blocks**	**Mean difference**	***t***	***p***	***d***
Consistent feedback	Signal	2 vs. 10	0.104	0.684	0.508	0.275
		10 vs. 15	0.000	0.000	1.000	0.000
		2 vs. 15	0.104	0.834	0.422	0.334
	Noise	2 vs. 10	0.313	3.362	0.006	1.241
		10 vs. 15	−0.042	−0.297	0.772	0.120
		2 vs. 15	0.271	1.711	0.115	0.670
Biased feedback	Signal	2 vs. 10	0.042	0.616	0.551	0.247
		10 vs. 15	−0.833	−0.771	0.457	0.309
		2 vs. 15	−0.042	−0.411	0.689	0.166
	Noise	2 vs. 10	0.292	2.88	0.015	1.077
		10 vs. 15	−0.104	−1.449	0.175	0.572
		2 vs. 15	0.188	2.283	0.043	0.877

### 4.3. Discussion

The mean accuracy in Experiment 3 was lower than that in Experiment 2. Since the accuracy in Experiment 2 referred to small-offset verniers, there was no case in which the high accuracy of large-offset verniers increased the overall accuracy in that experiment. Therefore, the change in accuracy could be attributed only to the difference in vernier categories. In Experiment 2, subjects were asked to distinguish between small and large offset verniers. In Experiment 3, they were asked to distinguish between small-offset and aligned verniers. The latter were more similar, which reduced their discriminability. In other words, the classification of stimuli in encoding learning depends on the discrimination of different categories. When there was no large-offset vernier as the guidance, even a small amount of biased feedback was enough to change the performance. This is why the accuracy decreased in the low group. In Experiments 2 and 3, the feedback was not completely consistent with the internal feedback of the subjects, with more or less randomness. For example, when a subject reported “4,” the actual feedback might be “3.” Conversely, feedback of “5” might be a “7.” In any case, such a difference prompted the subjects to doubt the internal feedback and finally change their decision.

In addition, the fact that the accuracy of noise changed more than that of the signal indicated that noise produced more false alarms. This might reflect a decision-making mechanism: compared to the internal feedback of the signal, which was visible, the internal feedback of noise was less clear and more easily susceptible to external feedback.

## 5. General discussion

### 5.1. Biased feedback decreases performance

Aberg and Herzog (2012) found no effect of biased feedback. The main difference in our experiments was that we asked participants to evaluate their performance, which was not included in the experiments of Aberg and Herzog (2012). Participants may not have clear internal feedback in their study. Thus, biased feedback influences perceptual judgment due to the conflict between internal and external feedback. This effect may come from encoding learning. According to the logic of Herzog and Fahle (1999), if decision learning occurs, all verniers will be influenced. In other words, in Experiments 1 and 2, if decision learning occurs, the subjects should make more “right” responses, and the accuracy of the left large-offset verniers should also decrease. Herzog and Fahle (1999) found a decision-learning effect: the performance of the large-offset verniers decreased in the first seven blocks and rebounded in the last five blocks, even though these verniers were easy to distinguish. However, our results supported the hypothesis about encoding learning in that subjects made more incorrect responses only regarding small-offset verniers. This implies that, at least when there is guidance, the subjects classify the small-offset verniers as right offset verniers rather than making more “right” responses to all the left offset verniers to pursue “good” feedback.

### 5.2. The mechanism of biased feedback

Feedback influences performance through a Hopfield network (Hertz et al., 1991). Learning without feedback has only two links: input and output. However, in a Hopfield network, the output will be fed back to the input. When feedback appears, the network begins to cycle. The feedback from the former trial (block) forms a feedforward network to the stimuli in the latter trials (blocks), which urges individuals to constantly revise their hypotheses to the task. The cycle of block feedback is relatively slow, while the cycle of trial feedback is faster. In a Hopfield network, the learning process can be seen as a transition from external feedback to internal monitoring (Bultena et al., 2017). When the revision of hypotheses meets the external feedback, the influence of the feedback reaches a peak, and the performance greatly improves. However, the consistency of internal and external feedback is an ideal state. In reality, external feedback is often compared with multiple internal standards (Latham and Locke, 1991), such as previous expectations (Kluger et al., 1994) and past performance levels (Carver and Scheier, 1990). Thus, the conflict between internal and external feedback seems irreparable, and the ideal state can rarely be reached.

The reversion of standards comes from the task-learning process described by Kluger and DeNisi (1996). They divided the feedback process into three levels: meta-task processes involving the self, task-motivation processes involving the focal task, and task-learning processes involving the task details of the focal task. In task-motivation processes, the preferred strategy to eliminate feedback and standard differences is to change behavior. When individuals are confronted with subjective failure that they want to overcome, they first try to work harder. If working harder does not lead to success, individuals may attempt to work smarter by finding alternative strategies to improve their performance, such as generating hypotheses regarding potential solutions (Wood and Locke, 1990). However, the mere motivation to learn may backfire because the more varied and elaborate attempts at the task (i.e., decreased cognitive consistency) are often futile.

Moreover, if the external feedback is not accompanied by cues that help to reject erroneous hypotheses, it may cause the recipient to generate a multitude of hypotheses that can reduce consistency and hence decrease performance (Kluger and DeNisi, 1996). Therefore, for a direct learning effect, the cues must be sufficient to help the recipient reject erroneous hypotheses. Unfortunately, biased feedback provides invalid cues rather than valid ones, so the subjects' efforts in the process of task learning do not improve their performance.

A worse situation is that, after a period of feedback cycling, the subjects may adjust their standards to a lower level. Loops that are high in the hierarchy can supervise the performance of lower-level loops, such that the output of higher-level loops may be the change of goals for lower-level loops (Kluger and DeNisi, 1996). For example, biased feedback may reduce self-efficacy. Low self-efficacy is the result of a high-level cycle (referring to the meta-task processes). This may lead to lower levels of task standards. In specific tasks, subjects may have lower requirements for the whole task and be more careless when performing it. Kanfer and Ackerman (1989) found that cognitive resources allocated to the external pressure to perform (attention to meta-task processes) may debilitate performance. Turning attention to high-level (meta-task process) or low-level (task-learning process) activities in the hierarchy may also be a factor when biased feedback decreases performance.

### 5.3. The monitoring of internal feedback

For most tasks, internal feedback exists. Individuals can judge their own performance and therefore serve as their own source of feedback (Ilgen et al., 1979). Herzog and Fahle (1999) also proposed that, because of the fast rebound toward the original performance, internal criteria must be involved in the discrimination process. If no internal criteria were involved, adjusting the decision criteria after correcting feedback should be as slow as adjusting to incorrect feedback during the first part of their experiments. In their study, the rebound in accuracy was accompanied by true feedback. However, in our experiments, accuracy rebounded when there was no feedback. Therefore, even without correcting external feedback, internal feedback is enough to help individuals monitor the task process and finally transfer their goal from obtaining “good” feedback to fulfilling the task requirements.

It should be noted that an important factor in the transfer of task objectives is whether there is a clear target classification. We found the rebound phenomenon in Experiment 2, but it did not appear in Experiment 3. This may be because the target classification is clear in Experiment 2 but fuzzy in Experiment 3. When the feedback disappears, the individual monitors the task based on internal feedback. A clear difference helps individuals recall the task goal in time, but a fuzzy difference makes them “lost” in the hypothesis created by biased feedback and has a lasting and irreversible impact.

There is no doubt that internal feedback exists not only in the stage when external feedback disappears but also in the stage when external feedback exists. Another related question is whether internal or external feedback is more important to individuals. The results indicated that when there was external feedback, the subjects were always easily influenced by it. Even if the medium-offset verniers in Experiment 1 were very easy to identify, the accuracy decreased. This may be related to the individual's attention to the two kinds of feedback. Compared with internal feedback, external feedback is unlikely to be ignored because any external feedback has potentially serious implications for the self. Since external feedback receives considerable attention, it has the capacity to alter the locus of attention. Therefore, in a Hopfield loop, external feedback has a greater weight. In addition, external feedback may reduce the weight of internal feedback (Sterzer et al., 2008; Summerfield et al., 2008). Although some researchers found that individuals believe that feedback from themselves is more important than feedback from others (Greller and Herold, 1975), this did not occur in the present studies. Instead, subjects appeared to be more susceptible to external feedback. Finally, for internal feedback, trial feedback and block feedback are different. In trial feedback, subjects can often form internal feedback on the details of each trial. When they perceive verniers, internal feedback is generated; otherwise, they cannot decide. However, individuals would not automatically form internal feedback to a set of trials (a block). That is why Aberg and Herzog (2012) found no block feedback effect since they did not ask the subjects to evaluate their performances. However, once individuals make an evaluation, regardless of how the small cycle between trials changes, the large cycle between blocks will also be influenced by the conflict between internal and external feedback. It is worth considering whether subjects will actively evaluate their performance on more complex tasks to improve their performance.

### 5.4. The generalization of false decisions

Why did the decrease in accuracy in Experiment 3 come from the incorrect judgment of noises? This was also related to the stimuli. In Experiments 1 and 2, the subjects had resolute confidence in the large offset condition. Therefore, when they found a conflict between internal and external feedback, the best strategy was to change the responses of the small-offset verniers. However, in Experiment 3, they may have been more confident in the small-offset verniers because the offsets were more or less visible, so they tried to change their judgment of the noises to meet the external feedback. This was consistent with the phenomenon called “overfitting” proposed by Hertz et al. (1991).

It should be noted that the definition of signal and noise in Experiment 3 is not a posteriori. In the introduction, we required the subjects to judge whether the two verniers were offset. The positive condition of offset was more easily defined as a signal. The different results of Experiments 2 and 3 indicate that the influence of biased feedback may partly depend on the discrimination of stimuli.

### 5.5. Limitation

To avoid subjects finding the feedback to be the opposite, we mixed true and biased feedback with a different probability. If there were a baseline of true feedback, the results would be more convincing. The conclusion that biased feedback influences perceptual judgment due to the conflict between internal and external feedback was based on the present study's results and Aberg and Herzog (2012). Testing how the difference between evaluation and feedback influences performance would be more convincing, but since the feedback was randomly generated, our program did not record the feedback.

The present study (particularly Experiment 3) raised some new questions. The first question is whether biased feedback has a cross-task influence. Biased feedback may have an impact through two paths: the meta-task process and the task-learning process. In this study, the tasks of all experiments were the same from the first block to the last block. If two different tasks are performed in the biased feedback blocks and rebound blocks, will the biased feedback still have an impact? If there is no cross-task influence, it implies that the meta-task process is not the reason why biased feedback influences performance. The second question is that in Experiment 3 when there was no large offset vernier, the role of biased feedback was very different from that in Experiment 2. We followed the design of Herzog and Fahle (1999) and regarded the large offset vernier as guidance. Will this help the subjects separate the task requirements? That is, in Experiments 1 and 2, the judgment of small (medium)-offset verniers was regarded as a task condition, while the judgment of large-offset verniers was regarded as another task condition. Finally, in the present study, after the end of the last feedback block, the subjects were told that there would be no feedback in the later blocks. If there is no such warning, would the rebound accuracy slow down?

We mentioned self-efficacy in 5.2, which is a typical factor of individual differences. Another factor that is more likely to interact with biased feedback is cognitive style. Individuals with greater field independence may be less affected by biased feedback, while field-dependent individuals may be more affected. The present study did not test the influence of individual differences. This could be a future direction of research. Another limitation is that even though we used the large-offset verniers as guidance, perhaps there were still some subjects who responded oppositely. It is difficult to distinguish whether their decision criteria have changed or their perceptions have changed. This can be further studied. Finally, self-assessment of performance may be related to memory, especially meta-memory. Biased feedback on memory is also a potential field for further study.

## 6. Conclusion

In summary, the present study's findings can be divided into four parts: (A) When biased feedback occurs, either trial feedback or block feedback negatively influences perceptual judgment. However, block feedback requires subjects to produce internal feedback based on the block cycle. (B) When the biased feedback disappears, the individual's perceptual judgment ability will rebound, which (at least in part) comes from the internal monitoring of the task. (C) If the difference between the two types of stimuli is large, encoding rather than decision-making learning will occur. (D) If the discrimination between the two types of stimuli is low, the individual's discrimination of noise is more vulnerable than that of the signal under the influence of biased feedback.

## Data availability statement

The original contributions presented in the study are publicly available. This data can be found here: https://doi.org/10.17632/6c7v9mhxj4.1.

## Ethics statement

The studies involving human participants were reviewed and approved by Ethics Committee of the Ministry of education of Guangxi Normal University. The patients/participants provided their written informed consent to participate in this study.

## Author contributions

CY designed the project and wrote the manuscript. ZX analyzed the data and reviewed the relevant literature. YZ was involved in revising the manuscript. TW revised the manuscript and provided equipment support. All authors contributed to the article and approved the submitted version.

## References

[B1] Aberg K. C. Herzog M. H. (2012). Different types of feedback change decision criterion and sensitivity differently in perceptual learning. J. Vis. 12, 3. 10.1167/12.3.3 22396463

[B2] Adams J. A. (1978). Theoretical issues for knowledge of results. Inform. Process. Motor Control Learn. 1978, 229–240. 10.1016/b978-0-12-665960-3.50016-5

[B3] Alloy L. B. Abramson L. Y. (1979). Judgment of contingency in depressed and nondepressed students: sadder but wiser? J. Exp. Psychol. Gen. 108, 441–485. 10.1037/0096-3445.108.4.441 528910

[B4] Arps G. F. (1920). Work with knowledge of results versus work without knowledge of results: awareness and partial awareness as factors conditioning efficiency. Psychol. Monogr. 28, i−41. 10.1037/h0093152

[B5] Beedie C. J. Lane A. M. Wilson M. G. (2012). A possible role for emotion and emotion regulation in physiological responses to false performance feedback in 10 mile laboratory cycling. Appl. Psychophysiol. Biofeedback 37, 269–277. 10.1007/s10484-012-9200-7 22752648

[B6] Book W. F. Norvell L. (1922). The will to learn an experimental study of incentives in learning. Pedagog. Semin. 29, 305–362. 10.1080/08919402.1922.10532882

[B7] Brown F. J. (1932). Knowledge of results as an incentive in school room practice. J. Educ. Psychol. 23, 532–552. 10.1037/h0074392

[B8] Bultena S. Danielmeier C. Bekkering H. Lemhöfer K. (2017). Electrophysiological correlates of error monitoring and feedback processing in second language learning. Front. Hum. Neurosci. 11, 29. 10.3389/fnhum.2017.00029 28194104 PMC5277024

[B9] Carver C. S. Scheier M. F. (1990). Origins and functions of positive and negative affect: a control-process view. Psychol. Rev. 97, 19–35. 10.1037/0033-295X.97.1.19

[B10] Chwilla D. J. Brunia C. H. (1991). Event-related potentials to different feedback stimuli. Psychophysiology 28, 123–132. 10.1111/j.1469-8986.1991.tb00400.x 1946878

[B11] Elwell J. L. Grindley G. C. (1938). The effect of knowledge of results on learning and performance. I. A coordinated movement of the two hands. Br. J. Psychol. 29, 39–53. 10.1111/j.2044-8295.1938.tb00899.x

[B12] Gilliland A. R. (1925). The effect of practice with and without knowledge of results in grading handwriting. J. Educ. Psychol. 16, 532–536. 10.1037/h0075734

[B13] Gray M. A. Harrison N. A. Wiens S. Critchley H. D. (2007). Modulation of emotional appraisal by false physiological feedback during fMRI. PLoS ONE 2, e546. 10.1371/journal.pone.0000546 17579718 PMC1890305

[B14] Greller M. M. Herold D. M. (1975). Sources of feedback: a preliminary investigation. Organ. Behav. Hum. Perform. 13, 244–256. 10.1016/0030-5073(75)90048-3

[B15] Hertz J. Krogh A. Palmer R. G. Horner H. (1991). Introduction to the theory of neural computation. Phys. Today 44, 70. 10.1063/1.2810360

[B16] Herzog M. H. Fahle M. (1999). Effects of biased feedback on learning and deciding in a vernier discrimination task. Vis. Res. 39, 4232–4243. 10.1016/S0042-6989(99)00138-8 10755160

[B17] Hill J. D. Salzman J. A. (2012). Enhancing speed perception in virtual environments through training. Proc. Hum. Fact. Ergonom. Soc. Ann. Meet. 56, 1772–1776. 10.1177/107118131256135625550444

[B18] Hurlock E. B. (1925). An evaluation of certain incentives used in school work. J. Educ. Psychol. 16, 145–159. 10.1037/h0067716

[B19] Ilgen D. R. Fisher C. D. Taylor M. S. (1979). Consequences of individual feedback on behavior in organizations. J. Appl. Psychol. 64, 349–371. 10.1037/0021-9010.64.4.349

[B20] Kanfer R. Ackerman P. L. (1989). Motivation and cognitive abilities: an integrative/aptitude-treatment interaction approach to skill acquisition. J. Appl. Psychol. 74, 657–690. 10.1037/0021-9010.74.4.657

[B21] Katz B. Jaeggi S. Buschkuehl M. Stegman A. Shah P. (2014). Differential effect of motivational features on training improvements in school-based cognitive training. Front. Hum. Neurosci. 8, 242. 10.3389/fnhum.2014.00242 24795603 PMC4006056

[B22] Kluger A. N. DeNisi A. (1996). The effects of feedback interventions on performance: a historical review, a meta-analysis, and a preliminary feedback intervention theory. Psychol. Bull. 119, 254–284. 10.1037/0033-2909.119.2.254

[B23] Kluger A. N. Lewinsohn S. Aiello J. R. (1994). The influence of feedback on mood: linear effects on pleasantness and curvilinear effects on arousal. Organ. Behav. Hum. Decis. Process. 60, 276–299. 10.1006/obhd.1994.1084

[B24] Latham G. P. Locke E. A. (1991). Self-regulation through goal setting. Organ. Behav. Hum. Decis. Process. 50, 212–247. 10.1016/0749-5978(91)90021-K

[B25] Locke E. A. (1967). Motivational effects of knowledge of results: knowledge or goal setting? J. Appl. Psychol. 51, 324–329. 10.1037/h0024771 6075575

[B26] Locke E. A. Bryan J. F. (1969). Knowledge of score and goal level as determinants of work rate. J. Appl. Psychol. 53, 59–65. 10.1037/h0026736

[B27] Manzer C. W. (1935). The effect of knowledge of output on muscular work. J. Exp. Psychol. 18, 80–90. 10.1037/h0057862

[B28] McCall K. M. Meston C. M. (2007). The effects of false positive and false negative physiological feedback on sexual arousal: a comparison of women with or without sexual arousal disorder. Arch. Sex. Behav. 36, 518–530. 10.1007/s10508-006-9140-5 17333325

[B29] Molloy O. Molesworth B. R. C. Williamson A. (2018). Improving young drivers' speed management behaviour through feedback: a cognitive training intervention. Transport. Res. Part F Traff. Psychol. Behav. 54, 324–337. 10.1016/j.trf.2018.02.010

[B30] Phillips G. C. Jones G. E. Rieger E. J. Snell J. B. (1999). Effects of the presentation of false heart-rate feedback on the performance of two common heartbeat-detection tasks. Psychophysiology 36, 504–510. 10.1017/S0048577299980071 10432800

[B31] Rigoni D. Braem S. Pourtois G. Brass M. (2016). Fake feedback on pain tolerance impacts proactive versus reactive control strategies. Conscious. Cogn. 42, 366–373. 10.1016/j.concog.2016.04.015 27149180

[B32] Schwark J. Sandry J. MacDonald J. Dolgov I. (2012). False feedback increases detection of low-prevalence targets in visual search. Attent. Percept. Psychophys. 74, 1583–1589. 10.3758/s13414-012-0354-4 22864899

[B33] Sterzer P. Frith C. Petrovic P. (2008). Believing is seeing: expectations alter visual awareness. Curr. Biol. 18, R697–R698. 10.1016/j.cub.2008.06.021 18727901

[B34] Story T. J. Craske M. G. (2008). Responses to false physiological feedback in individuals with panic attacks and elevated anxiety sensitivity. Behav. Res. Ther. 46, 1001–1008. 10.1016/j.brat.2008.06.001 18692167

[B35] Summerfield C. Trittschuh E. H. Monti J. M. Mesulam M.-M. Egner T. (2008). Neural repetition suppression reflects fulfilled perceptual expectations. Nat. Neurosci. 11, 9. 10.1038/nn.2163 19160497 PMC2747248

[B36] Tarullo A. R. Nayak S. St. John A. M. Doan S. N. (2018). Performance effects of reward-related feedback on the dimensional change card sort task. J. Genet. Psychol. 179, 171–175. 10.1080/00221325.2018.1466264 29757113

[B37] Thorndike E. L. (1927). The law of effect. Am. J. Psychol. 39, 212–222. 10.2307/1415413

[B38] Varrier R. S. Stuke H. Guggenmos M. Sterzer P. (2019). Sustained effects of corrupted feedback on perceptual inference. Sci. Rep. 9, 1. 10.1038/s41598-019-41954-z 30940859 PMC6445092

[B39] Wolfe J. M. Van Wert M. J. (2010). Varying target prevalence reveals two dissociable decision criteria in visual search. Curr. Biol. 20, 121–124. 10.1016/j.cub.2009.11.066 20079642 PMC2818748

[B40] Wood R. Locke E. A. (1990). Goal setting and strategy effects on complex tasks. Res. Organ. Behav. 12, 73–110.

[B41] Wright W. (1906). Some effects of incentives on work and fatigue. Psychol. Rev. 13, 23–34. 10.1037/h0076041

